# The Effects of Hyperuricemia on the Prognosis of IgA Nephropathy are More Potent in Females

**DOI:** 10.3390/jcm9010176

**Published:** 2020-01-08

**Authors:** Tae Ryom Oh, Hong Sang Choi, Chang Seong Kim, Kyung Pyo Kang, Young Joo Kwon, Sung Gyun Kim, Seong Kwon Ma, Soo Wan Kim, Eun Hui Bae

**Affiliations:** 1 Department of Internal Medicine, Chonnam National University Medical School, 42 Jebongro, Gwangju 61469, Korea; tryeomoh@daum.net (T.R.O.); hongsang38@hanmail.net (H.S.C.); laminion@hanmail.net (C.S.K.); drmsk@hanmail.net (S.K.M.); 2 Department of Internal Medicine and Research Institute of Clinical Medicine of Chonbuk National University-Chonbuk National University Hospital, Chonbuk National University Medical School, Jeonju 54907, Korea; kpkang@jbnu.ac.kr; 3 Department of Internal Medicine, Korea University College of Medicine, Seoul 02841, Korea; yjkwon@korea.ac.kr; 4 Department of Internal Medicine, Hallym University Sacred Heart Hospital, Anyang 14068, Korea; sgkim@hallym.ac.kr

**Keywords:** Ig A nephropathy, hyperuricemia, uric acid, glomerulonephritis, renal outcome, female, disease progression, prognosis, risk factor, sex

## Abstract

Hyperuricemia is a potential risk factor for immunoglobulin A nephropathy (IgAN) progression but its sex-specific effects on IgAN progression remain unclear. This study aimed to determine the effect of serum uric acid on IgAN progression and whether its effect varied according to sex. A total of 4339 patients who diagnosed with IgAN by renal biopsy were retrospectively analyzed. We assessed the association of serum uric acid on IgAN progression using Kaplan–Meier survival analyses and Cox proportional hazards models. The study’s primary end point was IgAN progression that was defined as a 50% decline in the estimated glomerular filtration rate or the initiation of dialysis. On average, the serum uric acid levels were higher in the men than in the women. In the fully adjusted Cox proportional hazards model that considered all subjects, the risk of IgAN progression increased by about 25.6% for every 1 mg/dL increase in the baseline uric acid level. The serum uric acid level was an independent risk factor for IgAN progression in both sexes but its effect was more pronounced in the women (hazard ratio [HR], 1.383; confidence interval [CI],1.263 to 1.514; *p *< 0.001) than in the men (HR, 1.181; CI, 1.097 to 1.272; *p* < 0.001) (*p*_interaction_ < 0.001). A sensitivity analysis involving serum uric acid quartiles generated consistent and robust results. In conclusion, the serum uric acid level was an independent risk factor for IgAN progression and its effect was more pronounced among the women compared with that among the men.

## 1. Introduction

In Korea, the prevalence and incidence of end-stage renal disease (ESRD) are increasing, and chronic glomerulonephritis (GN) accounts for approximately 8% of new ESRD diagnoses [1]. The progression of GN to chronic kidney disease (CKD) or ESRD is not uncommon and it generates a considerable socioeconomic burden [2]. Thus, timely GN diagnoses and early intervention are important for patients’ prognoses and socioeconomics.

Immunoglobulin A nephropathy (IgAN) is the most common type of primary GN in Korea and worldwide [3]. Identifying modifiable risk factors associated with IgAN progression is important, because the 30-year renal survival rate for IgAN is poor at 50.3% [4]. The traditional risk factors associated with IgAN progression include elevated serum creatinine levels, hypertension, and proteinuria at diagnosis [5]. Moreover, smoking [6], hyperuricemia [7], hyperlipidemia [7], and genetic factors [8,9] are associated with IgAN progression.

Hyperuricemia is an atherosclerotic factor [10,11], and evidence is accumulating that highlights the role of serum uric acid (UA) in hypertension, cardiovascular disease, and CKD [12,13,14]. Recently, several studies’ findings have shown that hyperuricemia is associated with IgAN progression but small numbers of patients were analyzed in these studies [7,15]. Recently, Fan S. et al. [16] also reported that hyperuricemia was associated with renal pathological findings such as segmental glomerulosclerosis, tubular atrophy, and interstital fibrosis in IgAN. In addition, Masukuma, Y. et al. [17] and Nagasawa, Y. et al. [18] have reported that the serum UA level is associated with IgAN progression and has a stronger relationship in women than in men. This study aimed to determine the effect of the serum UA level on IgAN progression and whether its effect varied according to sex.

## 2. Methods

### 2.1. Data Source and Study Population

**Figure 1:**
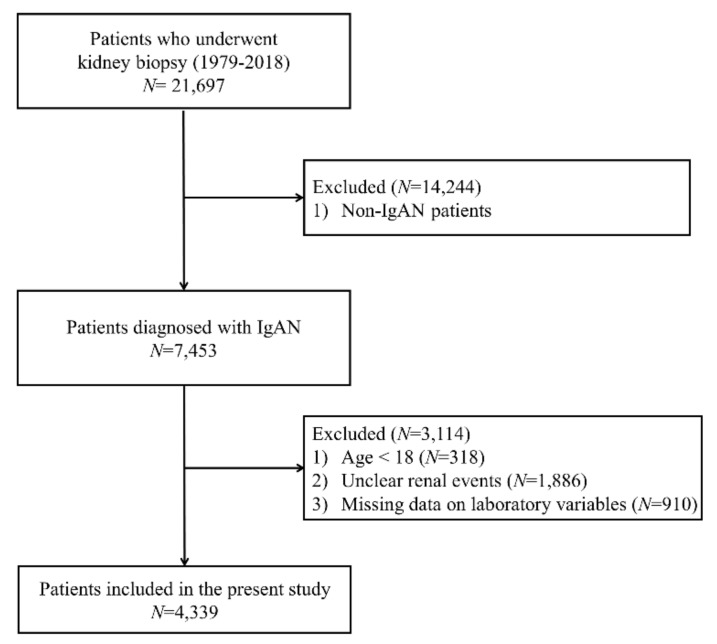
Flow diagram for patient’s enrollment. Abbreviation: IgAN, immunoglobulin A nephropathy.

Except for cancer patients and patients who previously received kidney transplants, 7453 of the 21,697 patients who underwent kidney biopsies at 18 university hospitals (Kyungpook National University Hospital, Kyung Hee University Hospital at Gandong, Kangdong Sacred Heart Hospital, Gangnam Severance Hospital, Korea University Guro Hospital, Korea University Anam Hospital, Eulji University Hospital, Seoul National University Boramae Medical Center, Seoul National University Bundang Hospital, Seoul National University Hospital, Severance Hospital, Pusan National University Yangsan Hospital, The Catholic University of Korea, Eunpyeong St. Mary’s Hospital, Ewha Womans University Mokdong Hospital, National Health Insurance Service Ilsan Hospital, Chonnam National University Hospital, Chonbuk National University Hospital, and Hallym University Sacred Heart Hospital) in Korea from January 1979 to October 2018 were diagnosed with IgAN. We excluded 318 patients aged <18 years, 1886 patients who did not know whether renal events had occurred, and 910 patients whose serum UA levels were not measured. Finally, the data from 4339 patients were retrospectively analyzed in this study. Figure 1 shows the flowchart for selecting cases for this study.

### 2.2. Study End Point, Definitions, and Measurements

The study’s primary end point was a major adverse renal event, namely, IgAN progression, which was defined as a 50% decline in the estimated glomerular filtration rate (eGFR) or the initiation of dialysis. Anemia was defined as a hemoglobin level <13 g/dL in the men and <12 g/dL in the women [19]. In our study, hyperuricemia was defined as a serum UA level >6 mg/dL in the men and >5 mg/dL in the women. The serum creatinine levels were measured using traceable isotope-dilution mass spectrometry. The eGFRs were calculated using the Modification of Diet in Renal Disease study equation [20].

### 2.3. Statistical Analyses

Regarding the continuous variables, the normally distributed data are expressed as the means and the standard deviations (SDs) and the skewed data are expressed as the medians and the interquartile ranges (IQRs) (25th percentile; 75th percentile). The Shapiro–Wilk test was used to assess the normality of the data. To compare the groups’ clinical characteristics and identify differences between them, Student’s t-test was used to analyze the normally distributed data and the Mann–Whitney U test was used to analyze the skewed data. The categorical variables are expressed as numbers and percentages and the chi-squared test was used to compare the groups regarding these variables. We used listwise deletion for missing data because <10% of the data were missing for all of the variables included in the present study except urine protein-to-creatinine ratio (UPCR). The entire study population was classified into quartiles based on the serum UA levels for the sensitivity analyses. Kaplan–Meier survival curves with log-rank tests and a univariate Cox proportional hazards model were used to examine the effect of the serum UA level on IgAN progression. A multivariate Cox proportional hazards model was applied to adjust the variables that may affect IgAN progression. Collinearity was analyzed to assess the variables’ interactions with the other independent variables. The proportional hazards assumption of the Cox proportional hazards model was verified using the Schoenfeld residuals test and log-minus-log survival plots. The hazard ratios (HRs) and 95% confidence intervals (CIs) were calculated to compare the risks of IgAN progression. Restricted cubic splines were used to demonstrate the non-linear relationships between the serum UA level and IgAN progression. The data were analyzed and plotted using R, version 3.5.2 (The R Foundation for Statistical Computing, Vienna, Austria; https://www.R-project.org/) [21]. All of the statistical tests were two-tailed and a value of *p* < 0.05 was considered statistically significant.

### 2.4. Ethics Approval and Consent to Participate

This study complied with the tenets of the Declaration of Helsinki. As the database used in this study did not include personal identifiers and the study was retrospective and observational in its design, the need for informed consent was waived. The study was approved by Chonnam National University Hospital’s Institutional Review Board (CNUH-EXP-2019-265).0

## 3. Results

### 3.1. Clinical Characteristics of the Study Population

**Figure 2:**
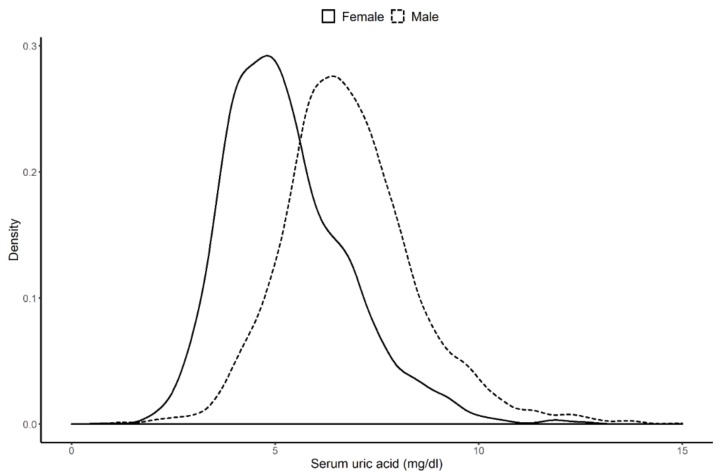
Difference in distribution of serum uric acid by sex. The median of serum uric acid level was 6.7 mg/dl in male and 5.0 mg/dl in female.

The data from a total of 4339 patients were analyzed. The study’s median (IQR) follow-up period was 6.1 years (2.7–9.8 years). The study population’s mean ± SD age was 39.3 ± 14.1 years, 50.5% of the patients were women, 7.6% of the patients had diabetes mellitus (DM), the median (IQR) eGFR was 75.1 mL/min.1.73 m^2^ (54.1–95.8 mL/min.1.73 m^2^) at baseline, and the mean ± SD systolic blood pressure was 123.9 ± 16.5 mmHg at baseline. Of the 4339 patients studied, 2525 (58.12%) had hyperuricemia based on their laboratory findings at biopsy. Table 1 summarizes the clinical characteristics of the control and hyperuricemia groups. Compared with the control group, there were more men and older people present in the hyperuricemia group. Compared with the control group, the hyperuricemia group had a higher prevalence of DM, a higher UPCR, and a lower eGFR. With the exception of the UPCR, <10% of the data were missing for all of the variables included in the present study. Figure 2 illustrates the distributions of the serum UA levels that differed significantly according to sex.

### 3.2. Crude Analysis of the Association Between the Serum Uric Acid Levels and the Progression of Immunoglobulin A Nephropathy

**Figure 3:**
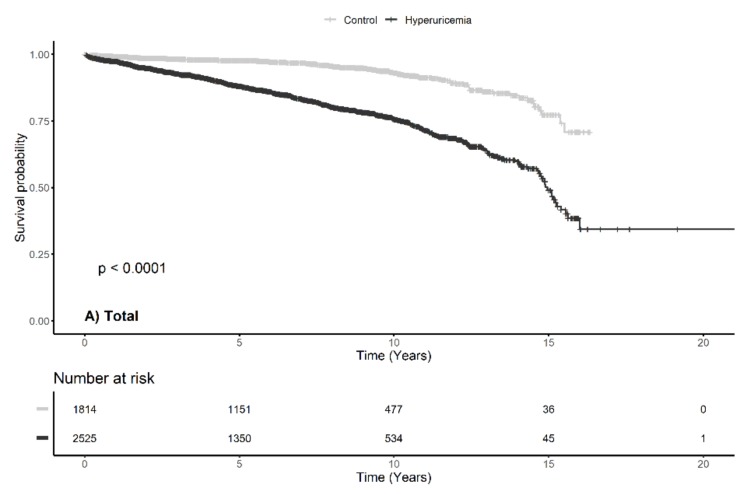

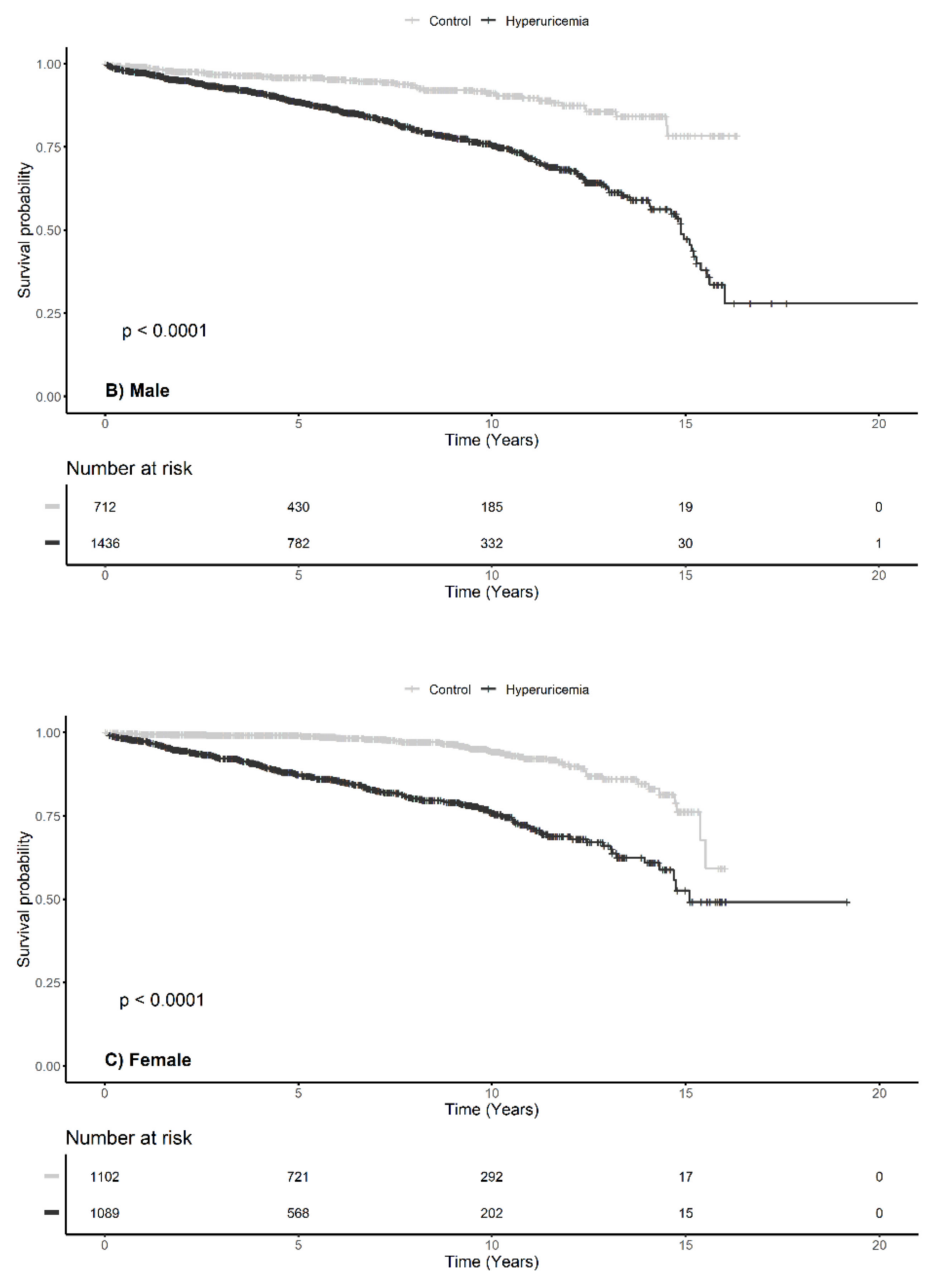
Kaplan—Meier survival curve with log-rank test between major adverse renal event and serum uric acid by sex.

A total of 568 (13.1%) patients experienced IgAN progression during the follow-up period; this occurred in 104 (5.7%) patients in the control group and in 464 (18.4%) patients in the hyperuricemia group (*p *< 0.001). Kaplan–Meier survival curves showed statistically significant differences between the hyperuricemia and the control groups in relation to IgAN progression when the entire study population was analyzed (Figure 3) and when the men and women were analyzed separately; IgAN progression occurred more frequently in the hyperuricemia group than in the control group.

**Figure 4:**
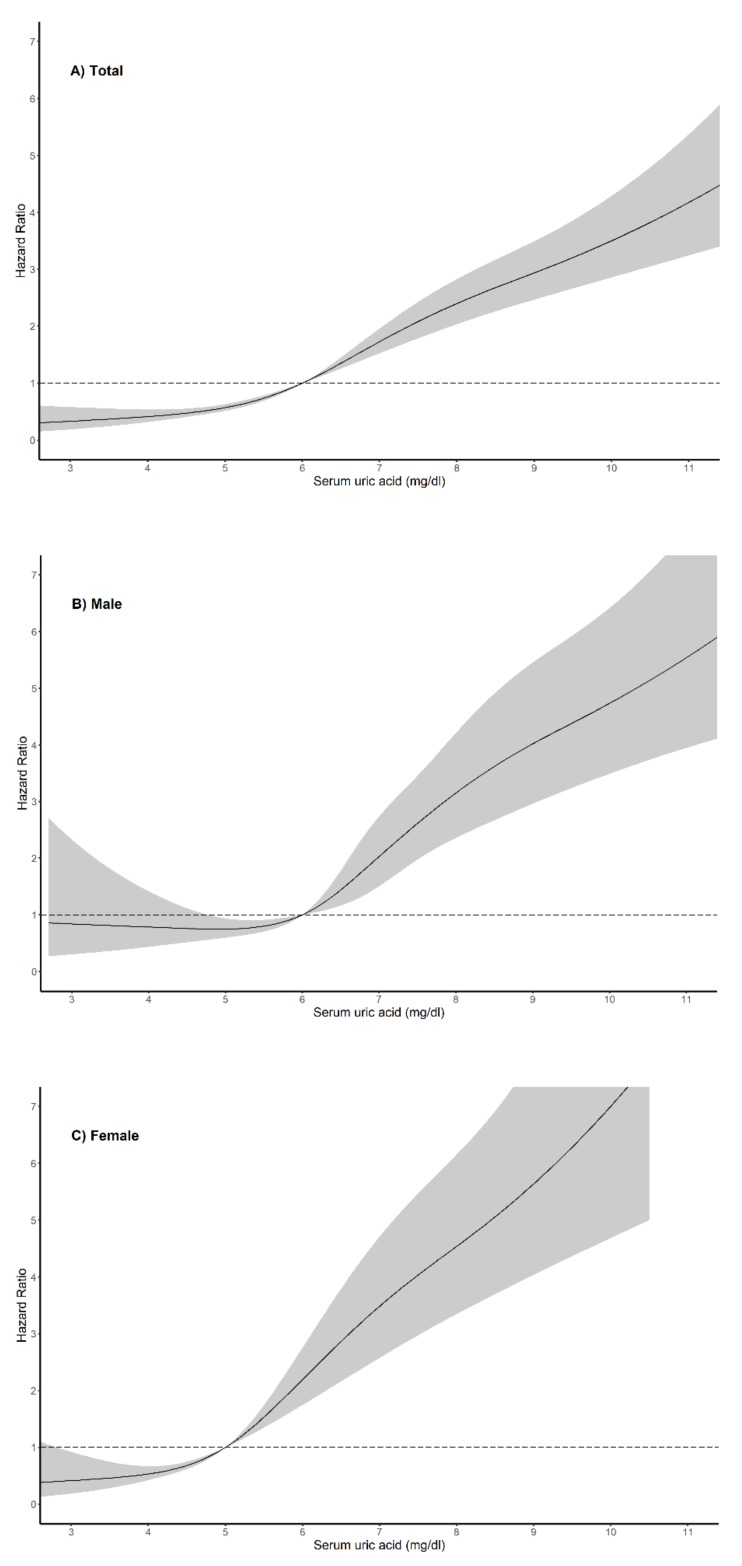
Restricted cubic spline curve of hazard ratio of serum uric for major adverse renal outcome by sex.

The results from the crude model of Cox proportional hazards analysis are presented in Table 2. Compared with the control group, the risk of IgAN progression was significantly higher among the patients with hyperuricemia (HR, 1.376; 95% CI, 1.329 to 1.425; *p *< 0.001). The association between the risk of IgAN progression and hyperuricemia was evident in the men (HR, 1.346; CI, 1.283 to 1.413) and in the women (HR, 1.514; 95% CI, 1.427 to 1.606) (*p*_interaction _< 0.001). Figure 4 shows the non-linear relationships between the serum UA level and IgAN progression in both sexes and within the entire study population.

Serum uric acid levels and major adverse renal outcome showed a nonlinear relationship in all groups.

### 3.3. Independent Risk Factors Associated with the Progression of Immunoglobulin A Nephropathy

Adjusted Cox proportional hazards models were used to determine whether serum UA was an independent risk factor for IgAN progression (Table 2). The analysis of the fully adjusted model that considered the entire study population showed that the risk of IgAN progression increased by approximately 25.6% for every 1 mg/dL increase in the baseline serum UA level (HR, 1.260; 95% CI, 1.181 to 1.332; *p *< 0.001). The results showed sex-specific difference when the men (HR, 1.188; 95% CI, 1.104 to 1.279; *p *< 0.001) and women (HR, 1.381; 95% CI, 1.259 to 1.513; *p *< 0.001) were analyzed separately (*p*_interaction_ < 0.001).

We performed Kaplan–Meier survival analyses and log-rank tests on the quartiles (Figure S1). The analyses of the entire study population and the men and women separately showed that quartile 4 had the lowest survival probability. As major adverse renal events can occur soon after biopsies for reasons other than disease progression, additional analyses performed on the patients excluded those who had a renal event during the first year post-biopsy, and the findings showed that serum UA was an independent risk factor associated with IgAN progression, regardless of sex (Table 3). Given that proteinuria is a well-known risk factor associated with renal survival, we included the UPCR in the adjusted Cox proportional hazards model (Table 3) and the analysis of the sex-specific Cox proportional hazards models generated HRs of 1.226 (95% CI, 1.135 to 1.325) for the men and 1.410 (95% CI, 1.279 to 1.553) for the women.

## 4. Discussion

The findings from this retrospective observational study of 4339 patients with IgAN showed that the serum UA level was an independent risk factor associated with IgAN progression. Further, hyperuricemia had effects on both sexes but its impact is greater on the women than on the men.

UA is the final purine oxidation metabolite in human beings and it is excreted in the urine mainly [22]. Its effects on the kidney are mediated via different biological mechanisms. Moreover, its antioxidant effect on the extracellular environment plays an important role in neurological diseases [23,24]. UA may help dendritic cells recognize apoptotic cells and it activates cluster of differentiation CD8^+^ cells in the immune system [25]. High intracellular UA concentrations cause proximal tubule dysfunction through the release of inflammatory chemokines [26]. Hyperuricemia may be involved in the mechanism underlying hypertensive vascular disease [27]. UA induces the production of monocyte chemoattractant protein-1 and platelet-derived growth factor in vascular smooth muscles cells [12,28]. Consequently, hyperuricemia may cause structural changes in the kidney, for example, arteriosclerosis, glomerulosclerosis, and tubular injuries, which eventually lead to irreversible disease [29]. Interestingly, an inverse correlation between the UA concentration and the renal blood flow has been reported [30].

While the relationship between hyperuricemia and IgAN has been clarified, differences between the sexes regarding this relationship were not analyzed in some studies [7,31,32]. Although studies of the sex-specific effects of serum UA levels have not generated conclusive results, serum UA tends to have a more powerful impact on women than men. Ben-Dov IZ et al. [33] analyzed the Jerusalem Lipid Research Clinic cohort data and found that serum uric acid is associated with all-cause mortality and may play a role of long-term predictor of acute kidney injury and CKD in both sexes. Barbieri et al. [34] reported that high serum UA levels were associated with severe coronary artery disease in women only. An analysis of 8285 patients showed that there was a statistically significant relationship between the serum UA level and coronary artery disease in women, and, particularly, among women aged ≥80 years [35]. Akasaka et al. [36] showed that elevated serum UA levels accelerated yearly eGFR declines and that women were more susceptible to this than men. In addition, among patients with CKD, the association between the serum UA level and cardiac hypertrophy was more pronounced among women than men [37]. Furthermore, significant associations were detected between the serum UA level and IgAN progression in women but not in men [17,18]; these findings are similar to the consistent and robust results from our study.

The reasons underlying the more potent effects of serum UA on women than on men are yet to be elucidated. Estrogen inhibits the function of urate transporter 1, reduces the serum UA level, and it promotes UA excretion in the urine [38]. Indeed, female hormones administered to postmenopausal women lowered their serum UA levels [39], and the serum UA level was higher in postmenopausal women as a consequence of reductions in the secretion of female hormones, which increased the risk of atherosclerosis [40,41]. Furthermore, renal arteriolar hyalinosis occurred at a lower serum UA concentration in women (≥5 mg/dL) compared with men (≥7 mg/dL) [42]. The serum UA concentration may be also related to glucose transporter 9 (GLUT9), which is encoded by the *SLC2A9* gene in human beings. *SLC2A9* is an important regulator of the serum UA level [43,44,45]. Variations in GLUT9 have a greater effect on women than on men and this accounted for 5–6% of the UA variance in women compared with 1–2% of the UA variance in men [43,46,47]. These findings support the hypothesis of sex-specific effects of serum UA but more research is needed to clarify the underlying mechanisms. Considering the results of these studies, screening for hyperuricemia in IgAN patients will help select a high-risk group of disease progression and appropriate interventions may have beneficial effects.

Although our study has many strengths, which include its large population and long follow-up period, the analyses have limitations. First, we could not prove causality between hyperuricemia and IgAN progression; the inability to prove causality is a trait of all observational studies. However, observational studies are powerful tools that enable assessments of epidemiologic relationships, and we capitalized on the complementary analytic methods to examine the relationship between the serum UA level and IgAN progression [48]. Second, as our study was retrospective and we used data from a registry, the results were susceptible to bias as a consequence of the overdiagnosis, underdiagnosis, or misclassification of patients. Third, our analyses could not resolve all of the problems associated with hidden biases and confounding factors. For example, the serum UA levels are affected by particular foods but data describing these foods were not available. If information about dietary habits had been analyzed in conjunction with the study’s current data, more robust conclusions would be possible. Fourth, our study lacked data that described the patients’ use of UA-lowering medications; the effects of these agents on the kidneys have emerged as a topic of considerable interest recently. As little information about drug use was available for this study, the analysis of the direct or indirect effects of UA-lowering agents on the kidneys was limited.

In conclusion, this study’s findings showed that the serum UA level was an independent risk factor for IgAN progression and its effect was more pronounced in the women than among the men.

## Figures and Tables

**Table 1:** Clinical characteristics of the subjects stratified by hyperuricemia.

Characteristics	Missing Data (*n* (%))	All Subjects (*n* = 4339)	Control (*n* = 1814)	Hyperuricemia (*n* = 2525)	*p*-Value
Age (year)	0 (0)	39.3 ± 14.1	37.9 ± 13.4	40.4 ± 14.5	<0.001
Male (%)	0 (0)	2148 (49.5)	712 (39.3%)	1436 (56.9)	<0.001
Height (cm)	341 (7.9)	164.9 [158.0; 172.0]	163.0 [157.4; 170.0]	166.0 [158.9; 173.0]	<0.001
Weight (kg)	193 (4.4)	63.6 [55.8; 72.5]	60.0 [53.6; 68.5]	66.1 [58.0; 75.0]	<0.001
Body mass index	355 (8.2)	23.4 [21.1; 26.0]	22.6 [20.7; 24.8]	24.0 [21.7; 26.6]	<0.001
Diabetes mellitus (%)	6 (0.1)	329 (7.6)	110 (6.1)	219 (8.7)	0.002
Systolic blood pressure (mmHg)	240 (5.5)	123.9 ± 16.5	120.4 ± 14.7	126.5 ± 17.2	<0.001
Diastolic blood pressure (mmHg)	241 (5.6)	77.4 ± 11.5	75.4 ± 10.8	78.9 ± 11.8	<0.001
Serum uric acid (mg/dl)	0 (0)	6.0 ± 1.8	4.5 ± 0.9	7.1 ± 1.4	<0.001
Hemoglobin (g/dl)	12 (0.3)	13.0 ± 1.9	12.9 ± 1.7	13.0 ± 2.0	0.301
Serum albumin (mg/dl)	18 (0.4)	3.9 [3.6; 4.2]	4.0 [3.7; 4.3]	3.9 [3.5; 4.2]	<0.001
Creatinine (mg/dl)	6 (0.1)	1.00 [0.80; 1.30]	0.84 [0.70; 1.00]	1.10 [0.90; 1.50]	<0.001
eGFR (ml/min/1.73 m^2^)	6 (0.1)	75.1 [54.4; 95.8]	86.4 [70.4; 104.5]	64.4 [44.4; 86.1]	<0.001
Total cholesterol (mg/dl)	242 (5.6)	184.0 [157.0; 215.0]	178.0 [155.0; 206.0]	189.0 [160.0; 220.0]	<0.001
Urine protein creatinine ratio (g/g Creatinine)	790 (18.2)	1.0 [0.5; 2.1]	0.8 [0.4; 1.6]	1.2 [0.6; 2.4]	<0.001
Follow-up duration (year)	0 (0)	6.1 [2.7; 9.8]	6.9 [3.2; 10.1]	5.5 [2.5; 9.4]	<0.001

Abbreviation: eGFR, estimated glomerular filtration rate by IDMS-MDRD equation.

**Table 2:** Hazard ratio of serum uric acid for major adverse renal event with Cox proportional hazard models by sex.

	Total Subjects		Male		Female	
	HR [95% CI]	*p*-Value	HR [95% CI]	*p*-Value	HR [95% CI]	*p*-Value
Crude	1.376 [1.329; 1.425]	<0.001	1.346 [1.283; 1.413]	<0.001	1.514 [1.427; 1.606]	<0.001
Model 1	1.398 [1.343; 1.447]	<0.001	1.338 [1.274; 1.404]	<0.001	1.507 [1.419; 1.600]	<0.001
Model 2	1.288 [1.227; 1.352]	<0.001	1.211 [1.134; 1.293]	<0.001	1.415 [1.316; 1.522]	<0.001
Model 3	1.260 [1.191; 1.332]	<0.001	1.188 [1.104; 1.279]	<0.001	1.381 [1.259; 1.513]	<0.001

Model 1, crude + age, sex (exclude sex in subgroup analysis). Model 2, Model 1 + creatinine, diastolic blood pressure. Model 3, Model 2 + anemia, body mass index, cholesterol, diabetes mellitus. Abbreviation: CI, confidence interval; HR, hazard ratio.

**Table 3:** Hazard ratio of serum uric acid for major adverse renal event with Cox proportional hazard models by sex with constraints.

Constraints	Total Subjects		Male		Female	
	HR [95% CI]	*p*-Value	HR [95% CI]	*p*-Value	HR [95% CI]	*p*-Value
Exclude first 1-year follow-up period ^†^						
Serum uric acid	1.298 [1.225; 1.377]	<0.001	1.235 [1.143; 1.333]	<0.001	1.405 [1.274; 1.550]	<0.001
Include urine protein creatinine ratio ‡						
Serum uric acid	1.228 [1.155; 1.306]	<0.001	1.151 [1.065; 1.245]	<0.001	1.380 [1.240; 1.533]	<0.001

^†^ Cox proportional model was adjusted with age, anemia, body mass index, cholesterol, creatinine, presence of diabetes mellitus, sex, serum uric acid and diastolic blood pressure. ‡ Cox proportional model was adjusted with age, anemia, body mass index, cholesterol, creatinine, presence of diabetes mellitus, sex, serum uric acid, diastolic blood pressure and urine protein creatinine ratio. Abbreviation: CI, confidence interval; HR, hazard ratio.

## Supplementary Materials

The following are available online at https://www.mdpi.com/2077-0383/9/1/176/s1. The supplementary includes that Kaplan–Meier survival analyses and log-rank tests on the quartiles.
