# Supplementary material for: The Effects of Hyperuricemia on the Prognosis of IgA Nephropathy are More Potent in Females
Source: J Clin Med. 2020 Jan 8;9(1):176. doi: 10.3390/jcm9010176 (PMC7019531; doi:10.3390/jcm9010176)
Supplement: Supplementary file 1 [file jcm-09-00176-s001.pdf]

The Effects of Hyperuricemia on the Prognosis of IgA Nephropathy are More Potent in Females

Tae Ryom Oh [1], Hong Sang Choi [1], Chang Seong Kim [1], Kyung Pyo Kang [2], Young Joo Kwon [3], Sung Gyun Kim [4], Seong Kwon Ma [1], Soo Wan Kim [1,*,†] and Eun Hui Bae [1,*,†]

1 Department of Internal Medicine, Chonnam National University Medical School, 42 Jebongro, Gwangju 61469, Korea; tryeomoh@daum.net (T.R.O.); hongsang38@hanmail.net (H.S.C.); laminion@hanmail.net (C.S.K.); drmsk@hanmail.net (S.K.M.)

2 Department of Internal Medicine and Research Institute of Clinical Medicine of Chonbuk National University-Chonbuk National University Hospital, Chonbuk National University Medical School, Jeonju 54907, Korea; kpkang@jbnu.ac.kr

3 Department of Internal Medicine, Korea University College of Medicine, Seoul 02841, Korea; yjkwon@korea.ac.kr

4 Department of Internal Medicine, Hallym University Sacred Heart Hospital, Anyang 14068, Korea; sgkim@hallym.ac.kr

* Correspondence: skimw@chonnam.ac.kr (S.W.K.); Tel.: +82-62-220-6271; baedak76@gmail.com (E.H.B.); Tel.: +82-62-220-6503, Fax: +82-62-225-8578

† These authors have contributed equally to this manuscript as correspondence authors.

Supplementary Materials

Figure S1. Kaplan-Meier survival curve with log-rank test for composite renal outcome by quartiles of uric acid

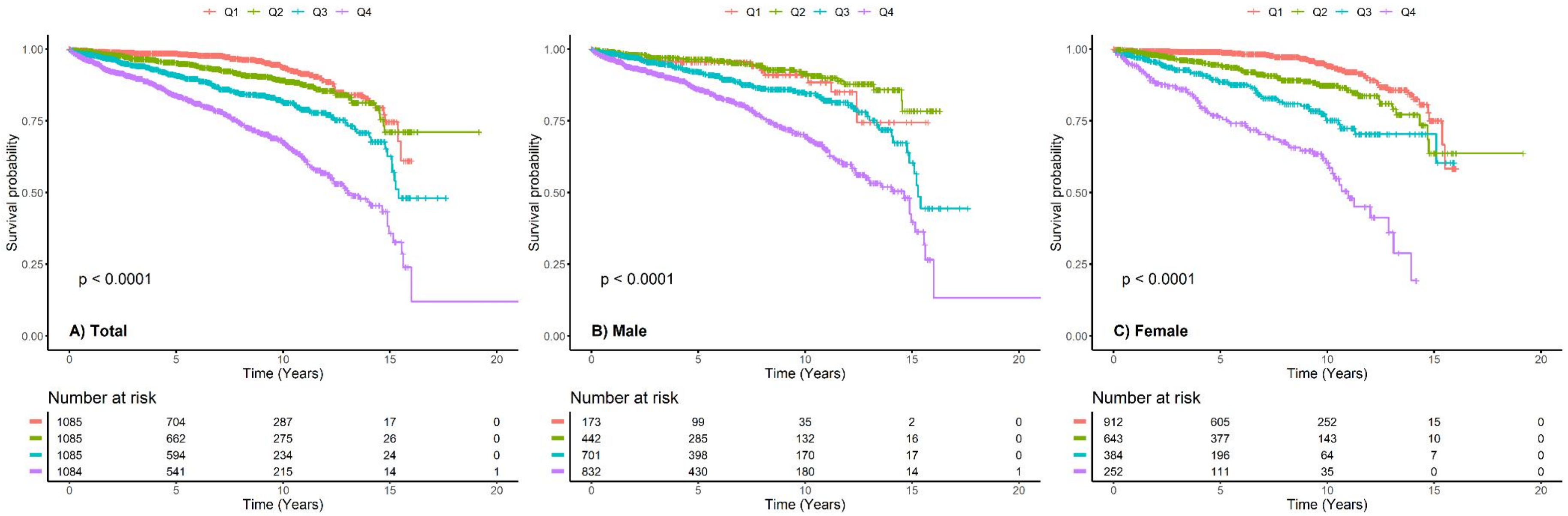

Quartiles 4 showed the highest event incidence in all groups.
